# Supplementary material for: Causal Relationship between Aging and Anorexia Nervosa: A White-Matter-Microstructure-Mediated Mendelian Randomization Analysis
Source: Biomedicines. 2024 Aug 16;12(8):1874. doi: 10.3390/biomedicines12081874 (PMC11351342; doi:10.3390/biomedicines12081874)
Supplement: Supplementary file 1 [file biomedicines-12-01874-s001.zip › Supplementary Figure/Figure S4/Singe SNP plot of MR analysis for TL to ubm-b-1849.pdf]

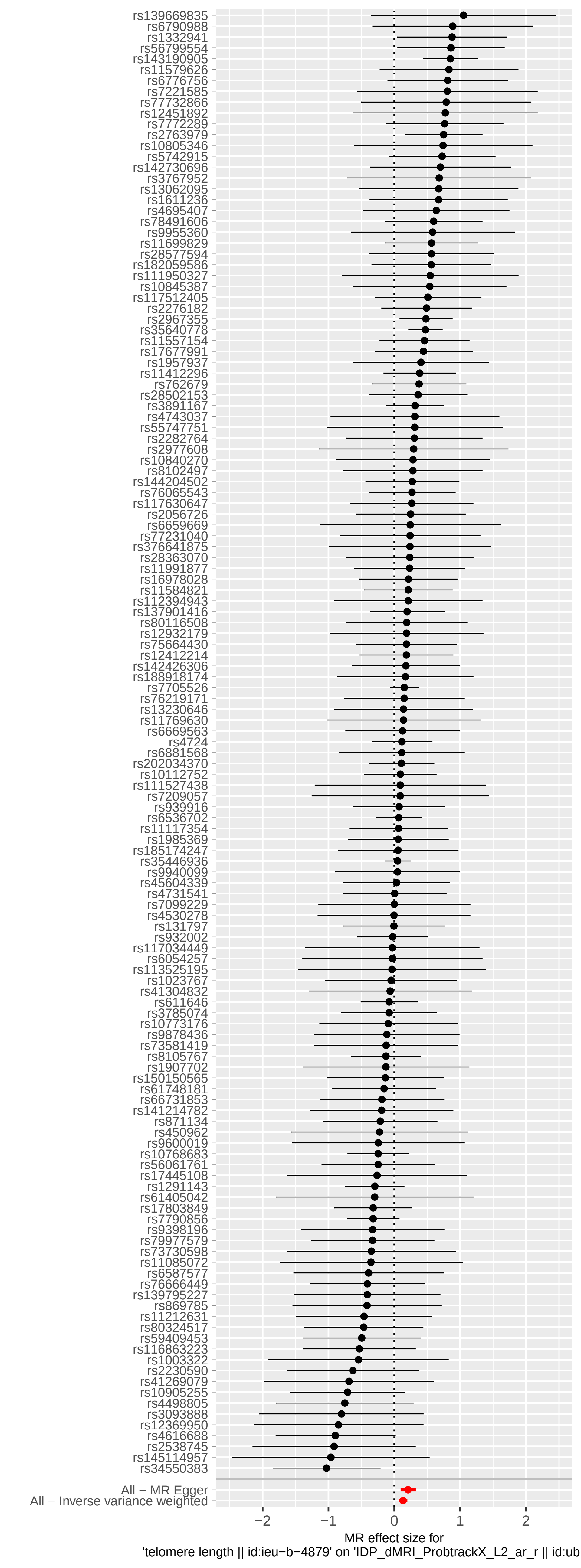
rs139669835
rs6790988
rs1332941
rs56799554
rs143190905
rs11579626
rs6776756
rs7221585
rs77732866
rs12451892
rs7772289
rs2763979
rs10805346
rs5742915
rs142730696
rs3767952
rs13062095
rs1611236
rs4695407
rs78491606
rs9955360
rs11699829
rs28577594
rs182059586
rs111950327
rs10845387
rs117512405
rs2276182
rs2967355
rs35640778
rs11557154
rs17677991
rs1957937
rs11412296
rs762679
rs28502153
rs3891167
rs4743037
rs55747751
rs2282764
rs2977608
rs10840270
rs8102497
rs144204502
rs76065543
rs117630647
rs2056726
rs6659669
rs77231040
rs376641875
rs28363070
rs11991877
rs16978028
rs11584821
rs112394943
rs137901416
rs80116508
rs12932179
rs75664430
rs12412214
rs142426306
rs188918174
rs7705526
rs76219171
rs13230646
rs11769630
rs6669563
rs4724
rs6881568
rs202034370
rs10112752
rs111527438
rs7209057
rs939916
rs6536702
rs11117354
rs1985369
rs185174247
rs35446936
rs9940099
rs45604339
rs4731541
rs7099229
rs4530278
rs131797
rs932002
rs117034449
rs6054257
rs113525195
rs1023767
rs41304832
rs611646
rs3785074
rs10773176
rs9878436
rs73581419
rs8105767
rs1907702
rs150150565
rs61748181
rs66731853
rs141214782
rs871134
rs450962
rs9600019
rs10768683
rs56061761
rs17445108
rs1291143
rs61405042
rs17803849
rs7790856
rs9398196
rs79977579
rs73730598
rs11085072
rs6587577
rs76666449
rs139795227
rs869785
rs11212631
rs80324517
rs59409453
rs116863223
rs1003322
rs2230590
rs41269079
rs10905255
rs4498805
rs3093888
rs12369950
rs4616688
rs2538745
rs145114957
rs34550383
All − MR Egger
All − Inverse variance weighted
−2
−1
0
1
2
MR effect size for
'telomere length || id:ieu−b−4879' on 'IDP_dMRI_ProbtrackX_L2_ar_r || id:ub
